# Is central dogma a global property of cellular information flow?

**DOI:** 10.3389/fphys.2012.00439

**Published:** 2012-11-23

**Authors:** Vincent Piras, Masaru Tomita, Kumar Selvarajoo

**Affiliations:** ^1^Institute for Advanced Biosciences, Keio UniversityTsuruoka, Yamagata, Japan; ^2^Graduate School of Media and Governance, Keio UniversityFujisawa, Kanagawa, Japan

**Keywords:** gene expression, central dogma, biological noise, correlation analysis, emergent behavior

## Abstract

The central dogma of molecular biology has come under scrutiny in recent years. Here, we reviewed high-throughput mRNA and protein expression data of *Escherichia coli, Saccharomyces cerevisiae*, and several mammalian cells. At both single cell and population scales, the statistical comparisons between the entire transcriptomes and proteomes show clear correlation structures. In contrast, the pair-wise correlations of single transcripts to proteins show nullity. These data suggest that the organizing structure guiding cellular processes is observed at omics-wide scale, and not at single molecule level. The central dogma, thus, globally emerges as an average integrated flow of cellular information.

Information processing is essential in all fields of science. In molecular biology, the central dogma, first coined by Francis Crick (Crick, 1958, 1970), is a classical backbone of living cells to fundamentally execute processes from cell division to death through the DNA, RNA, and protein information pathways. More specifically, the central dogma describes the transfer of sequence information during DNA replication, transcription into RNA, and translation into amino-acid chains forming proteins. At the same time, it also states that information cannot flow from protein to protein or nucleic acid.

Since the advent of systemic and high throughput approaches over the last two decades, these broad steps, which do not include complex regulatory details, have come under intense scrutiny. The missing regulatory features, such as the DNA proofreading/repair mechanisms and alternative splicing of pre-mRNA, introduce several intermediary steps. These additional steps interfere with the key steps of the dogma and likely alter the information dynamics. In addition, epigenetics, or the role played by chromatin structures, DNA methylation and histone modifications, also seem to go against the simple pathways of the dogma (Shapiro, 2009; Luco et al., 2011). Protein splicing, or the ability of a protein (inteins) to alter its own sequence, discovered in recent times (Volkmann and Mootz, 2012) and prions, which modify other protein sequences (Prusiner, 1998), bypass the information transfer pathway of the dogma. Other investigations reported errors or mismatches between RNA sequences and their coding DNA (Hayden, 2011; Li et al., 2011). Taken together, these data cast doubts on the validity of the central dogma in the context of present day science and, therefore, question the simplicity of linear information flow (DNA to RNA, and RNA to protein).

To put things into perspective, we require analytical tools that investigate the concerns or discrepancies regarding the long-standing theory. One simple, yet highly useful technique for searching global properties in high-throughput datasets is statistical correlation analysis, which has been widely and successfully used to observe patterns in complex systems such as the weather (Stewart, 1990), stock markets (Lo and MacKinlay, 1988) and cosmology (Amati et al., 2008). There are several kinds of correlation analyses that evaluate both linear (e.g., Pearson product-moment) and non-linear (e.g., Spearman's rank, Mutual Information) dependencies (Steuer et al., 2002; Rosner, 2011). In particular, the Pearson product-moment correlation analysis has become the most popular due to its ability to show organizational structure in the simplest form.

In biology, there have been numerous works that have studied the correlations in the mRNA and protein expression data (see below and Table 1). In theory, when two samples containing high-dimensional (such as microarray and proteomic) data are compared, the correlation analyses provide a measure of deviation from unity as a source of difference between the samples. Briefly, two samples with identical and completely non-identical information will show unit (*R*^2^ = 1) and null (*R*^2^ = 0) correlation, respectively.

**Table 1:** **mRNA and protein expression correlations in various organisms**.

**Organism**	***N***	***R*^2^**	**References**
**SINGLE CELL mRNA vs. PROTEIN**			
*Escherichia coli*	1	~0.01^a^	Taniguchi et al., 2010
**SINGLE CELL mRNA vs. mRNA**			
*Mus musculus* (Oocyte)	21,436	0.92^b^	Tang et al., 2009
*Homo sapiens* (HeLa)	~29,000	0.75–0.78	Fan et al., 2012
*Homo sapiens* (Brain tumor)	~29,000	0.70–0.77	Fan et al., 2012
**SINGLE CELL PROTEIN vs. PROTEIN**			
*Homo sapiens* (Macrophage)	12	~0.72^b^	Shin et al., 2011
**CELL POPULATION mRNA vs. PROTEIN**			
*Escherichia coli*	841	0.29	Taniguchi et al., 2010
	437	0.47	Lu et al., 2007
*Desulfovibrio vulgaris*	392–427	0.20–0.28	Nie et al., 2006
*Saccharomyces*	71	0.58	Futcher et al., 1999
*cerevisiae*	328	0.01^a^^,^^b^–0.36^b^	Fournier et al., 2010
*Schizosaccharomyces pombe*	1367	0.34	Schmidt et al., 2007
*Streptomyces coelicolor*	884	0.40	Jayapal et al., 2008
*Mus musculus* (NIH/3T3)	5028	0.31^b^–0.41	Schwanhäusser et al., 2011
*Homo sapiens* (Medulloblastoma)	511	0.22	de Sousa Abreu et al., 2009
**CELL POPULATION mRNA vs. mRNA**			
*Mycobacterium tuberculosis*	3989	0.9	Ward et al., 2008
*Mus musculus* (NIH/3T3)	5028	0.91^b^	Schwanhäusser et al., 2011
**CELL POPULATION PROTEIN vs. PROTEIN**			
*Porphyromonas gingivalis*	751	0.97–0.99	Xia et al., 2007
*Glycine max* (Soybean)	163–287	0.9	Brandão et al., 2010
*Mus musculus* (NIH/3T3)	5028	0.90^b^	Schwanhäusser et al., 2011

a Corresponding mutual information I ~ 0.

b Values we computed from raw data.

Perfect correlation (*R*^2^ = 1) is an idealized situation that is far from reality, as technical or experimental noise alone interferes and reduces correlation. Moreover, the recent years have highlighted the existence of biological noise: the studies on individual cells and molecules have shown stochasticity in gene expression dynamics due to the combinatorial effect of low molecular copy numbers and the quantal nature of promoter dynamics (Raj and van Oudenaarden, 2009; Eldar and Elowitz, 2010). On the other hand, clonal populations of cells display heterogeneity in the levels of a given protein expression per cell at any measured time (Chang et al., 2008). Together, stochasticity and heterogeneity are essential for producing cell fate diversification, phenotypic variations, and amplification of intracellular signals (Locke et al., 2011; Selvarajoo, 2012).

The stochastic fluctuations, or intrinsic noise, cause the expression of a molecular species to vary in time and between cells, leading to uncorrelated responses (Elowitz et al., 2002). This is especially prominent for mRNAs and proteins with low copy numbers. Thus, the between samples (cells) correlation can be lowered due to intrinsic noise (Figure 1A). Other sources of biological noise due to extrinsic factors include variability in cell size, molecular copy numbers, and environmental fluctuations between individual cells. These factors distort the deterministic central dogma and likely alter strong correlations into weaker ones (Figure 1B).

**Figure 1:**
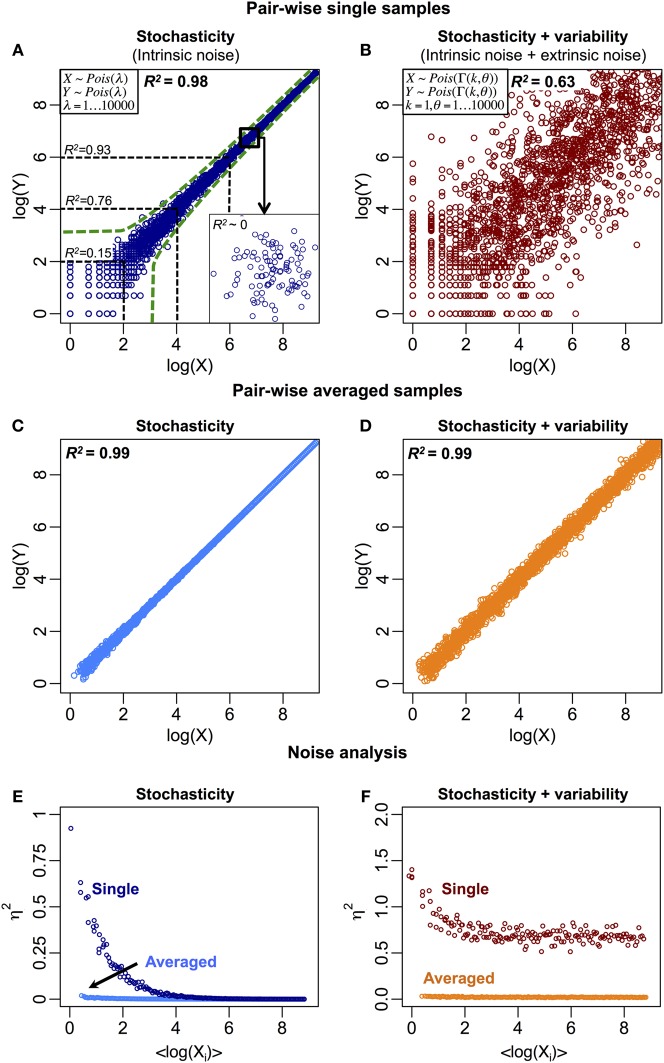
**Biological and non-biological noise reduce the between samples correlation structure. (A)** Stochastic fluctuations reduce correlations, especially for low copy number of molecular species (*R*^2^ ~0.15 for log(*X*) < 2). The green dotted lines represent the intrinsic noise region generated by Poisson process (Raj and van Oudenaarden, 2009). Insert: the correlation structure disappears when zooming at smaller or single molecule scale. **(B)** Stochastic fluctuations (intrinsic) on variable (extrinsic) noise further reduce the overall correlation structure. Variable noise is represented by a Gamma distribution (Taniguchi et al., 2010). *R*^2^ is obtained by squaring the Pearson product-moment correlation coefficient, $R=r(X,Y)=\sum_{i=1}^{N}\left(x_{i}-\mu_{X})(y_{i}-\mu_{Y}\right)/\sqrt{\sum_{i=1}^{N}{\left(x_{i}-\mu_{X}\right)}^{2}}\sqrt{\sum_{i=1}^{N}{\left(y_{i}-\mu_{Y}\right)}^{2}},$ where ***X*** = (*x*_1_, …, *x*_*i*_, …, *x*_*N*_) and ***Y*** = (*y*_1_, …, *y*_*i*_, …, *y*_*N*_) are 2 *N*-dimensional variables, *x*_*i*_ and *y*_*i*_ are the *i*th observation (*i* = 1, …, *N*) of ***X*** and ***Y*** respectively. μ_*X*_ and μ_*Y*_ are the statistical means of the two variables. **(C)** Stochastic and **(D)** total (stochastic and variable) noise reduce when single samples are averaged into population. (**E**) and **(F)** show noise, η^2^ = σ^2^_*XY*_/μ^2^_*XY*_, versus <log(*X*_*i*_)> for **(C)** and **(D)**, respectively, where $\sigma_{XY}=\sqrt{\frac{1}{2P}\sum_{j=1}^{P}{\left(x_{i,j}-y_{i,j}\right)}^{2}},\mu_{XY}=\frac{1}{2P}\sum_{j=1}^{P}\left(x_{i,j}-y_{i,j}\right)$ (Jones and Payne, 1997), and the *j*th element of vectors ***X***_*i*_ = (*x*_*i*, 1_, …, *x*_*i,j*_, …, *x*_*i,P*_) and ***Y***_*i*_ = (*y*_*i*, 1_, …, *y*_*i,j*_, …, *y*_*i,P*_) is the expression of the *i*th gene in the *j*th sample for (*P* = 100) pairs of samples. In **(F)**, at higher expressions for single cells, the remaining noise represents the extrinsic or variable noise. At averaged population scale, this noise is significantly reduced due to the effect of random noise cancellation.

One recent study compared *Escherichia coli* mRNA and protein expressions between individual cells at single molecule level and provided a scenario that deeply questions the central dogma. Taniguchi et al. (2010) revealed that there is no correlation (*R*^2^ ~ 0) between individual *tufA* mRNA and protein levels in single cells. Notably, they concluded that the lack of correlation is likely due to differences in mRNA and protein lifetimes. Although this is a plausible explanation, Taniguchi et al. were careful not to disprove the long-holding hypothesis by claiming that time averages of mRNA levels should correlate with protein levels. However, there was no evidence shown to demonstrate that this is the actual case, and when we evaluated non-linear dependencies using mutual information (Steuer et al., 2002; Tsuchiya et al., 2010) in Taniguchi et al. dataset, we found the result to be non-dependent, i.e., *I* ~ 0. This confirms that mRNA to protein expressions between individual cells at single molecule level are clearly unrelated. Furthermore, when zooming at single molecule level in the correlation plot, it is evident that their pair-wise correlations are weak (Figure 1A, insert, for illustration).

Notably, at cell population level, Taniguchi et al. were able to show relatively high correlation between mRNA and protein expressions with *R*^2^ = 0.29 (Figure 2A). In fact, another independent study by Lu et al. (2007), for *E. coli* population, also showed relatively high correlation (*R*^2^ = 0.47). Similar analyses performed on *Saccharomyces cerevisiae* (Futcher et al., 1999), murine NIH/3T3 fibroblast (Schwanhäusser et al., 2011) and several other cell populations (Nie et al., 2006; Schmidt et al., 2007; Jayapal et al., 2008; de Sousa Abreu et al., 2009) all showed correlated structures between transcriptome-wide and proteome-wide expressions (Table 1). So, why is there no correlation between individual mRNA and protein expressions in single cells, while at population level, collective relationships are observed between large-scale mRNA and protein expressions?

**Figure 2:**
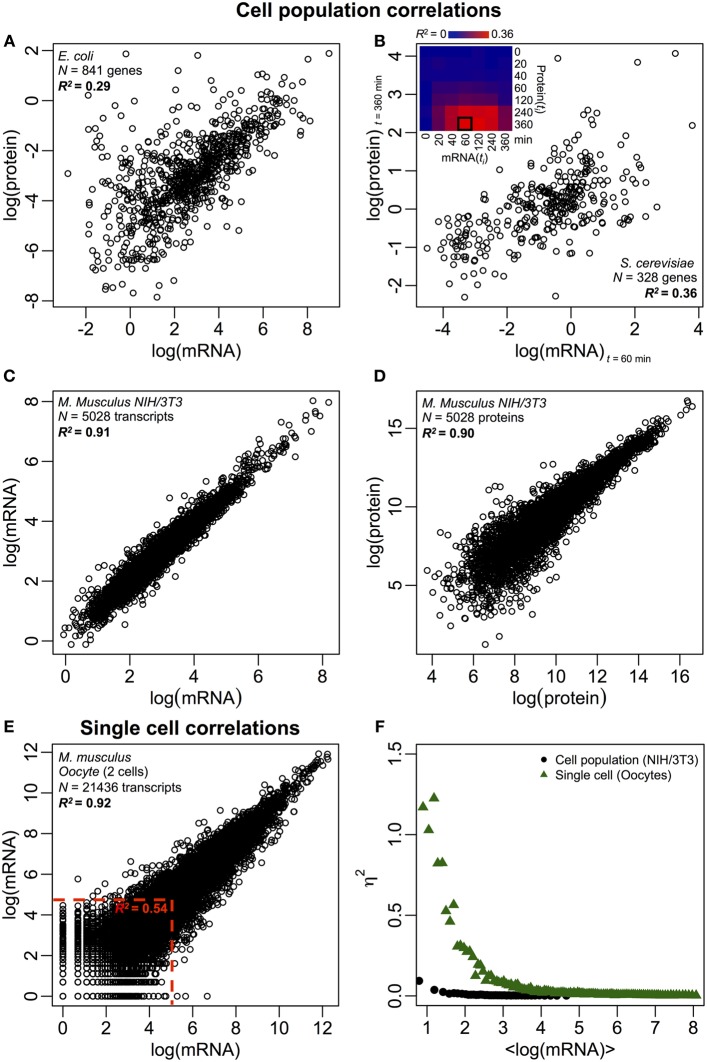
**Omics-wide expression correlations.** Cell populations: mRNA-protein correlations in **(A)** *E. coli* (Taniguchi et al., 2010) and **(B)** *S. cerevisiae* (Fournier et al., 2010) between mRNA expressions at *t* = 60 min and protein expressions at *t* = 360 min. Insert: correlation matrix between all time points shows a delayed increase in correlations between mRNA and proteins. **(C)** mRNA and **(D)** protein expressions between two samples of murine NIH/3T3 cells (Schwanhäusser et al., 2011). Single cells: **(E)** mRNA expressions between two oocytes (Tang et al., 2009). The red dotted lines indicate the regions of low mRNA expressions (log(mRNA) < 5). **(F)** Noise (η^2^) versus log(mRNA expressions) for cell population (NIH/3T3, black dots, Schwanhäusser et al., 2011) and single cells (Oocytes, green triangles, Tang et al., 2009). Each dot represents the value for a group of *P* = 100 mRNAs. η^2^ is near zero for the cell population for all mRNA expressions. For single cells, η^2^ is highest for mRNAs with the lowest copy numbers, and approaches zero for higher copy numbers.

We believe there are two major reasons for the differences. Firstly, as noted earlier, noise, whether biological or non-biological in nature, reduces correlation. Since analyses on single cells have shown the importance of stochasticity and variability, these effects are crucial for reducing single cell correlations. At ensemble level, when cells are sampled into a population, the total (intrinsic + extrinsic) noise is reduced, as random noise cancels out across all range of molecular expressions (Figures 1C–F), to reveal average response and self-organization (Karsenti, 2008; Selvarajoo, 2011; Hekstra and Leibler, 2012; Selvarajoo and Giuliani, 2012). Hence, a good degree of mRNA-protein expression correlation emerges. Secondly, for the single cell study (Taniguchi et al., 2010), individual mRNA-protein expression correlation was compared across numerous cells. In cell population studies, however, the comparison is made in entirety, across thousands of mRNAs and proteins over several orders of magnitude greater than the range of expression found for single molecule between cells. This, therefore, leads to higher correlations at population level as the effect of single molecular variations becomes negligible.

Despite correlated structures being observed for cell populations, there are tangible reasons for the large deviation from perfect correlation. As noted earlier, one key point is that mRNAs and proteins are sequentially located with several missing processes, unrepresented in the central dogma. Adding the missing intermediates along a biochemical pathway will incur a noticeable delay in information flow (Selvarajoo, 2006, 2011; Piras et al., 2011), and the correlation between them could suffer as a result. This could also be part of the fact noted by Taniguchi et al. that mRNA and protein expressions have different lifetimes. Notably, this postulation is supported in a recent work on *S. cerevisiae* treated with Rapamycin that showed the temporal correlations of mRNA-protein expression were initially low, *R*^2^ = 0.01 at 40 min, nevertheless, over 360 min after perturbation, the correlation increased, *R*^2^ = 0.36 (Fournier et al., 2010, Figure 2B). The data indicate that upon chemical perturbation, the initial response between mRNA and protein expressions deviates due to time-delay and different kinetic mechanisms between them, as well as secondary effects such as autocrine or paracrine signaling interference (Shvartsman et al., 2002; Isalan et al., 2008). When the effects of the perturbation are attenuated over time, the recovery of correlations occurred.

To further check the postulation that sequential delay processes or different lifetimes are crucial for decreasing mRNA-protein correlations, we compared *R*^2^ between the same molecular species of the central dogma (e.g., between mRNA and mRNA) in cell populations and single cells. The transcriptome-wide mRNA-mRNA expression correlation between replicates of NIH/3T3 (Schwanhäusser et al., 2011) (Figure 2C) and *Mycobacterium tuberculosis* (Ward et al., 2008) cell population samples are both very high, with *R*^2^ > 0.9 (Table 1). Such strong correlations are also observed between population samples for protein–protein expressions in NIH/3T3 cells (Schwanhäusser et al., 2011) (Figure 2D), *Porphyromonas gingivalis* (Xia et al., 2007) and *Glycine max* (Brandão et al., 2010) (Table 1). Since these data that compare same species yield very high correlations, it is conceivable that the sequential delay processes or different lifetimes are responsible for lowering the population level correlation structures between mRNA and protein expressions.

In single murine oocytes (Tang et al., 2009), when comparing entire mRNA–mRNA expressions, a highly correlated structure is observed (*R*^2^ = 0.92, Figure 2E). However, focusing only on lowly expressed mRNAs (with logarithmic expressions < 5), the stochastic noise lowers the pair-wise correlation quite dramatically (*R*^2^ < 0.54). To probe this result we evaluated noise, η^2^ = σ^2^_*XY*_/μ^2^_*XY*_, across entire mRNA expressions (Figure 2F). We noted that η^2^ is highest for the lowest expressions, due to the pronounced effect of stochastic fluctuations in comparison to their expressions, and approaches zero for higher expressions, where such noise becomes less significant (Piras et al., 2012). For cell population, as expected, near zero noise is observed across the entire expression range due to the canceling out of random noise (Figures 1E,F).

Highly correlated structures for entire mRNA–mRNA expressions were also reported for single cancer cell (Fan et al., 2012), albeit less significant with *R*^2^ ~ 0.7 (Table 1). Furthermore, protein–protein expressions comparison in LPS-stimulated human macrophages also showed high correlations, *R*^2^ ~0.72 (Shin et al., 2011) (Table 1). Although there is no correlation between individual mRNA-protein expressions in single cells, the large-scale or omics-wide correlation between same molecular species in single cells is very high.

Thus, whether single cells or cell populations, the omics-wide data indicate that the correlations between the same molecular species (mRNA vs. mRNA, and protein vs. protein) are noticeably higher than between different species (mRNA vs. protein). This reflects the fact that although time-delay processes and differing lifetimes are key for reducing correlations, these mechanisms are not sufficient for supporting the lack of correlation structure observed between single cells' individual transcript to protein expressions.

So far, through investigating large-scale expressions of mRNAs and proteins of various cellular systems, we have shown that correlation structures emerge at a global scale. However, the correlation analyses reveal only the connectivity between two tested samples, and do not show the direction of information flow. For the central dogma to be valid on a global scale, the overall flow of information should be from DNA to proteins. Such flow of information has been demonstrated by myriad other studies that involve perturbing the receptors of cell populations and monitoring the resultant dynamics of transcription factors binding to DNA and the induction of large scale gene expressions (Figure 3A). For example, in the case of LPS-stimulated immune cells, it has been demonstrated that the activation of the transcription factor NF-κB occurs at around 15 min (Liu et al., 1999), the induction of its downstream genes at about 30 min (Liu et al., 1999; Xaus et al., 2000; Selvarajoo et al., 2008), and the translation of the corresponding proteins in the region of 60–90 min (Kawai et al., 1999; Xaus et al., 2000) (Figure 3B). Such sequential direction of the overall transcription to translation information flow is also observed for bacterial systems, such as *E. coli*, at cell population level (Golding et al., 2005).

**Figure 3:**
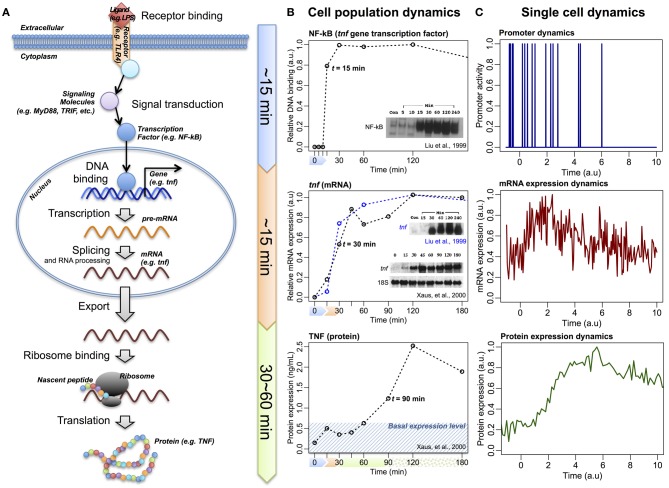
**The information flow of central dogma. (A)** Schematic of LPS/TLR4-induced TNF expression, via transcription factor NF-κB and *tnf* gene, following linear information flow. **(B)** Experimental temporal profiles of promoter binding activity of NF-κB (upper panels), *tnf* (middle panels), and TNF (lower panels) expressions at cell population level. **(C)** Schematic temporal profiles of promoter dynamics, mRNA, and protein expressions at single-cell level (Raj and van Oudenaarden, 2009).

Alternatively, investigations at single cell resolution reveal random fluctuations over the linear information flow: the transcription factors binding to DNA promoter regions is quantal, resulting in bursting behavior of the mRNA transcription and, subsequently, induces variability in the protein translation, even between identical cells (Figure 3C) (Raj and van Oudenaarden, 2009; Eldar and Elowitz, 2010; Locke et al., 2011; Hekstra and Leibler, 2012; Selvarajoo, 2012). As a result, at any particular time point, the individual molecular response for single cells is rather noisy compared to population average scale (Selvarajoo, 2011).

## Conclusions

The examples shown in this paper highlight the differences in the order of correlation values observed between species in the central dogma over cell populations and single cells. The statistical analyses from cell populations paint a picture that the expression correlation between the same molecular species is very high and between species is moderately high. Although single cell correlations between the same species are comparable with cell populations, they showed a wider scatter in their expressions plots due to the pronounced effect of biological noise, especially for transcripts with low copy numbers. Notably, the single cells' pair-wise correlation becomes zero for individual molecules (Taniguchi et al., 2010). In fact, stochastic fluctuations and variability in molecular expressions are known to be functional in generating cell fate decision and tipping cellular states (Losick and Desplan, 2008; Eldar and Elowitz, 2010; Kuwahara and Schwartz, 2012). We believe that the strong omics-wide correlations occur as a result of tight gene and protein regulatory networks across thousands of molecules (Barabási and Oltvai, 2004; Karsenti, 2008) resulting in emergent average responses. Analyzing small number or individual molecules, the correlation structure cannot be observed.

Overall, it is conceivable that viewing the information flow of single DNA to protein will question the central dogma as the response of each molecule at any single time will not likely correlate. However, globally, the observation of average deterministic response suggests that the net equilibrium of the genetic information remains to the far right of the pathways. Therefore, the central dogma should be viewed as a macroscopic cellular information flow on an omics-wide scale, and not at single gene to protein level. As such, we believe its simplicity will continue to remain as one of the most influential theoretical pillars of living systems.

### Conflict of interest statement

The authors declare that the research was conducted in the absence of any commercial or financial relationships that could be construed as a potential conflict of interest.
